# FTY720 Suppresses Liver Tumor Metastasis by Reducing the Population of Circulating Endothelial Progenitor Cells

**DOI:** 10.1371/journal.pone.0032380

**Published:** 2012-02-27

**Authors:** Chang Xian Li, Yan Shao, Kevin T. P. Ng, Xiao Bing Liu, Chang Chun Ling, Yuen Yuen Ma, Wei Geng, Sheung Tat Fan, Chung Mau Lo, Kwan Man

**Affiliations:** Department of Surgery, Centre for Cancer Research and State Key Laboratory of Liver Research,The University of Hong Kong, Hong Kong, China; Chinese University of Hong Kong, Hong Kong

## Abstract

**Background:**

Surgical procedures such as liver resection and liver transplantation are the first-line treatments for hepatocellular carcinoma (HCC) patients. However, the high incidence of tumor recurrence and metastasis after liver surgery remains a major problem. Recent studies have shown that hepatic ischemia-reperfusion (I/R) injury and endothelial progenitor cells (EPCs) contribute to tumor growth and metastasis. We aim to investigate the mechanism of FTY720, which was originally applied as an immunomodulator, on suppression of liver tumor metastasis after liver resection and partial hepatic I/R injury.

**Methodology/Principal Findings:**

An orthotopic liver tumor model in Buffalo rat was established using the hepatocellular carcinoma cell line McA-RH7777. Two weeks after orthotopic liver tumor implantation, the rats underwent liver resection for tumor-bearing lobe and partial hepatic I/R injury. FTY720 (2 mg/kg) was administered through the inferior caval vein before and after I/R injury. Blood samples were taken at days 0, 1, 3, 7, 14, 21 and 28 for detection of circulating EPCs (CD133+CD34+). Our results showed that intrahepatic and lung metastases were significantly inhibited together with less tumor angiogenesis by FTY720 treatment. The number of circulating EPCs was also significantly decreased by FTY720 treatment from day 7 to day 28. Hepatic gene expressions of CXCL10, VEGF, CXCR3, CXCR4 induced by hepatic I/R injury were down-regulated in the treatment group.

**Conclusions/Significance:**

FTY720 suppressed liver tumor metastasis after liver resection marred by hepatic I/R injury in a rat liver tumor model by attenuating hepatic I/R injury and reducing circulating EPCs.

## Introduction

Hepatocellular carcinoma (HCC) is one of the most common malignancies in the world [1]. Surgical procedures such as liver resection and liver transplantation are the first-line treatments for HCC patients. However, the high incidence of tumor recurrence and metastasis after liver surgery remains a major problem [2], [3]. Therefore, it is a pressing need to develop novel therapies to eliminate tumor recurrence and metastasis after liver surgery.

Surgical stress injury such as hepatic ischemia reperfusion (I/R) injury is an inevitable consequence during liver surgery. Hepatic I/R injury promote liver tumor growth and metastases through activation of cell adhesion, invasion, and angiogenesis pathways [2]–[4]. Furthermore, accumulating evidence indicated that surgical stress injury can rapidly increase the number of circulating EPC [5]–[7]. These events are also associated with elevated levels of vascular endothelial growth factor (VEGF), stem cell factor (SCF), and granulocyte colony-stimulating factor (G-CSF) that stimulate the release of EPCs from the bone marrow [5], [8]–[10].

EPCs, a subtype of progenitor cells in postnatal bone marrow, have the capacity to migrate to the peripheral circulation and differentiate into mature endothelial cells [11], [12]. Several researches have showed that circulating level of EPCs is higher in patients with advanced HCC, which may act as a potential prognostic marker in HCC patients [13], [14]. Furthermore, EPCs play important roles in tumor vasculogenesis and tumor growth at early phase by providing structural support to nascent vessels and the release of pro-angiogenic cytokines [10], [15]–[17]. EPCs have major roles in the tumor progression from micrometastases to macrometastases [18].

FTY720, is synthetically derived from myriocin (ISP-1), a metabolite isolated from ascomycete, Isaria sinclarii [19]. FTY720 has been demonstrated to attenuate hepatic I/R injury by ameliorating acute phase inflammatory response and up-regulating several protective genes including heat shock proteins and anti-apoptotic genes [20], [21]. Recently several groups have shown that FTY720 has a strong antitumor effect on liver cancer, breast cancer, bladder cancer, and prostate cancer [22]–[25]. Therefore, our hypothesis was that FTY720 may suppress liver tumor metastasis after liver surgery through attenuating hepatic I/R injury and subsequently reducing circulating EPCs.

In this study, we aimed to investigate whether FTY720 suppresses liver tumor metastasis after liver tumor resection and partial hepatic I/R injury by attenuating hepatic I/R injury and reducing circulating EPCs level in an orthotopic rat liver tumor model. The significance of this study will hopefully open a novel therapy to reduce liver tumor metastasis after liver surgery for HCC patients.

## Materials and Methods

### Animal Model

Buffalo rat hepatoma cell line McA-RH7777 (1×10^6^/100 ul) (Purchased from the American Type Culture Collection, Number CRL1601, ATCC, Manassas, VA, USA) labeled with luciferase [26], was injected into hepatic capsule of buffalo rat to induce solid tumor. When the size of tumor volume reached 10×10 mm (length×width) in size, tumor tissues were harvested and cut into 1–2 mm^3^ cubes and implanted into the left liver lobes of a new group of buffalo rats. Two weeks after orthotopic liver tumor implantation, the orthotopic liver tumor reached around 1 cm in diameter. The right branch of portal vein and hepatic artery were clamped for 30 minutes by micro vessel clamp and released afterwards. The left lobe of the liver was removed after cross clamping of the portal vein. Rats were housed in a standard animal laboratory with free activity and access to water and food. They were kept under constant environment conditions with a 12-hour light-dark cycle. All operations were performed under clean conditions. The study had been licensed according to Animal Ordinance Chapter 340 by the Department of Health, Hong Kong Special Administrative Region (ref.: (08-64) in DH/HA&P/8/2/3 Pt. 3).

### Treatment Regimen, Imaging Analysis, and Sample Collection

FTY720 (2 mg/kg, diluted with saline water, treatment group) or saline water (control group) were administrated through the inferior caval vein before and after I/R injury. Both the treatment group and the control group comprised 22 rats each. Six rats in each group were sacrificed at 6 h after reperfusion to study the role of FTY720 in attenuate hepatic I/R injury and expressions of some inflammatory cytokines and chemokines. Remaining Buffalo rats were used to monitor tumor metastasis after hepatectomy and hepatic I/R injury by the Xenogen *in vivo* imaging system through detection of luminance signals from tumor cells [27]. Blood samples were collected at days 0 (before liver resection and I/R injury), 1, 3, 7, 14, 21 and 28 from tail vein for detection of circulating EPCs. And then Buffalo rats were sacrificed at 4 weeks after liver resection and I/R injury. Liver, lung, kidney, and spleen suspected of tumor metastasis were sampled for further investigation. Furthermore, rat bone-marrow was also collected from rat femurs for detecting the number of EPCs.

### Detection of Circulating and Bone Marrow EPCs by Flow Cytometry

Rat peripheral blood mononuclear cells (PBMCs) were isolated after lysis red blood cells with a 1× ACK lysis buffer(NH_4_Cl 8.29 g/l,KHCO_3_ 1 g/l,Na_2_-EDTA 37.2 mg/l). PBMCs were recovered by spinning at 1000 rpm for 5 minutes and were washed extensively with PBS containing 1% BSA. Cells were re-suspended in PBS-1% BSA, counted, and recovered by centrifugation. For analysis of EPCs surface molecules, cells were stained with the following antibodies: unconjugated rabbit anti-CD133 (Abcam, Cambridge, UK), PE-conjugated anti-CD34 (Santa Cruz Biotech, Santa Cruz, CA), PE-Cy5-conjugated anti-CD45 (BD Pharmingen, San Diego, CA) and goat anti-rabbit FITC secondary antibody (Abcam, Cambridge, UK). Corresponding isotype-matched control monoclonal antibodies were used in all flow cytometric staining procedures. Flow cytometric analysis was performed using a FACSCalibur (Becton Dickinson, San Diego, CA).

### Hematoxylin and Eosin (H & E) and Immunohistochemical (IHC) Staining

The samples were fixed in 10% formalin and embedded in paraffin. Paraffin sections (4 µm thick) were dewaxed in xylene, rinsed in grade alcohol, and rehydrated in water and then was stained with H&E for histological examination. The expression of CD34 (Santa Cruz Biotechnology) was detected by immunohistochemical staining [27]. The first step was the same as the H&E staining, and then the paraffin sections placed in citric buffer (pH 6.0) and treated in a microwave. Afterwards, the sections underwent blocking with 10% FBS for 30 min. Subsequently, primary antibodies with proper dilution were applied on the sections, which were then incubated at 4°C overnight. Then the sections underwent blocking with 3% peroxidase for 30 min and following that, secondary antibodies from Dako EnVision System (DakoCytomation) were applied, and the sections were incubated for 30 min at room temperature. Signals were developed with 3,3′-diaminobenzidine substrate solution (DakoCytomation). The sections were finally counterstained by hematoxylin solution.

### Determination of microvessel density and Tumor Load Analyses

Microvessel density (MVD) of liver tumor tissue sections was evaluated [27], [28]. Any CD34+ stained endothelial cell or endothelial cell cluster that was clearly separated from adjacent microvessels, tumor cells, and connective elements was counted as one microvessel. The mean microvessel count of the six most vascular areas was taken as the MVD, which was expressed as the absolute number of microvessels per 1.485 mm2 (×200 field). Intrahepatic tumor load was scored as the percentage of hepatic replacement area (HRA) by tumor in liver based on 4 nonsequential hematoxylin-eosin staining sections by an integrated imaging system [29]. The number of lung metastasis tumor nodules was also counted.

### Detection of Hepatic Apoptosis by TUNEL Staining

TUNEL assay was performed to detect the apoptotic nuclei in paraffin sections, by using *in situ* Cell Death Detection Kit, POD (Roche Applied Science, Mannheim, Germany) [21].

### Assessment of Hepatic Gene Expression Profiles

Liver tumor tissue was stored at −80°C until total RNA extraction. Total RNA was extracted using RNeasy Midi kit (Qiagen). Total RNA (1 µg) from each sample was used to perform reverse transcription reaction using high capacity cDNA Kit (Applied Biosystems). Reverse transcription product (1 µl) was used to perform real-time quantitative reverse transcription-PCR by ABI PRISM 7700 Sequence Detection System (Applied Biosystems). Amplification of 18 S ribosomal RNA was applied as the internal control. Readings from each sample and its internal control were used to calculate gene expression level. After normalization with the internal control, gene expression levels were expressed as the folds relative to the normal liver. The sequences of the primers were listed as follows: VEGF: Left AAATGCTTTCTCCGCTCTGA, Right TTCCTGCAGCATAGCAGATG; IP10: Left TTCCAATCCCAGCTACATC, Right ACTCAGACTCAGCAGCAC; CXCR3: Left AGCACAGACACCTTCCTGCT, Right CAGAGACCAGAGCCGAAAAC; CXCR4: Left CTCCAAGCTGTCACACTCCA, Right GATGCTGATCCCCACGTAAT.

### Protein expression of ROCK, IP10, VEGF

Western blot was done with a modified version of a method described previously [30]. Anti-CXCL10, anti-VEGF, anti-CXCR3 and anti-CXCR4 antibodies were purchased from Santa Cruz Biotechnology.

### Statistics and Data Analyses

Continuous variables were expressed as median with range. Mann-Whitney U test was used for statistical comparison. χ^2^ test was used to compare incidence of intrahepatic and lung metastasis after hepatectomy and I/R injury. P<0.05 was considered statistically significant. Calculations were performed by using the SPSS computer software version 15 (SPSS, Chicago, IL).

## Results

### FTY720 suppressed liver tumor metastasis after major hepatectomy and partial hepatic I/R injury

**Figure 1:**
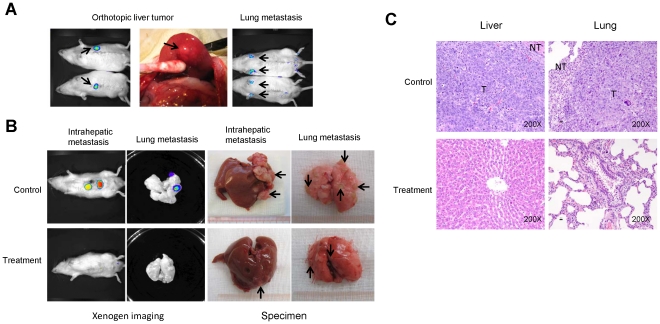
FTY720 suppressed liver tumor intrahepatic and lung metastasis after hepatectomy and I/R injury. (A) Orthotopic liver tumor model with metastatic potentials was established in buffalo rats. (B) intrahepatic and Lung metastasis were detected by Xenogen imaging and confirmed in specimen at four weeks after liver resection and I/R injury. (C) Histological features of liver tumors from buffalo rats at four weeks after hepatectomy and partial I/R injury with and without FTY720 treatment.

In order to investigate the effect of FTY720 on the metastasis of liver tumor after hepatectomy and partial I/R injury, we established a rat orthotopic liver tumor model with local and distant metastatic potentials (Figure 1A). Before the major hepatectomy and partial I/R injury, the average size of liver tumor did not show significant difference between the treatment and control group; the mean diameters of the tumors were 1.1±0.3 cm and 1.0±0.3 cm, respectively. The result showed that, after FTY720 treatment, the incidence of intrahepatic metastasis decreased from 68.75% (11 of 16) to 37.5% (6 of 16; P = 0.07) (Table 1). Similarly, there was an inhibition of lung metastasis by FTY720 treatment, manifested by a drop of incidence from 56.25% (9 of 16) to 31.25% (5 of 16; P = 0.15) (Table 1). More importantly, the number of metastatic tumor nodules in lung and metastatic tumor size in liver were significantly reduced by FTY720 treatment (Table 1). Intrahepatic and lung metastasis were detected by the Xenogen *in vivo* imaging system and further confirmed by histology examination (Figure 1B, C). Tumor metastasis was not found in other organs in both treatment and control group.

**Table 1:** Comparison of tumor metastasis after liver resection and I/R injury.

	Controlgroup	Treatmentgroup	P value
Intrahepatic metastasis			
Metastasis ratio	11/16 (68.75%)	6/16(37.5%)	0.07
HRA(% tumor in liver)	33.84% (15–45.6%)	19.11% (4.4–22.6%)	0.006
Lung metastasis			
Metastasis ratio	9/16 (56.25%)	5/16 (31.25%)	0.15
Number(tumor nodules)/rat	8.4(3–20)	3.6 (1–8)	0.046

Note: average (range).

### FTY720 significantly reduced circulating and bone marrow EPCs

**Figure 2:**
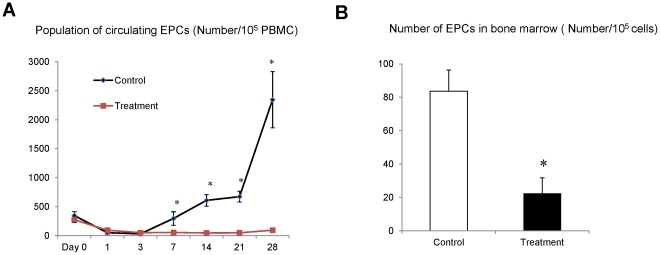
Comparison of circulating EPCs and bone marrow EPCs by Flow Cytometry. (A) The number of circulating EPCs at different time points after the major hepatectomy and partial I/R injury. (B) The number of EPCs in bone-marrow was detected by Flow Cytometry at four weeks later after liver resection and I/R injury. Each group contained 16 rats, *P<0.05.

**Figure 3:**
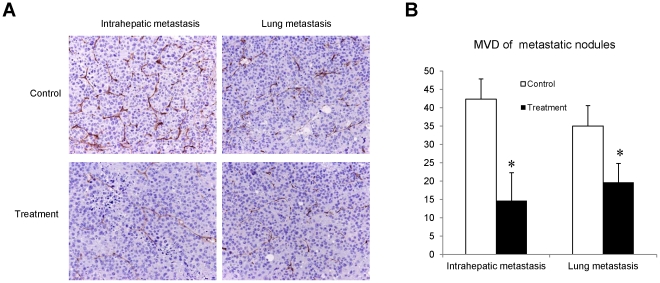
Comparison of tumor angiogenesis in metastatic tumor nodules. (A) The expression of CD34 positive cells in intrahepatic and lung metastatic tumor nodules. (B) Comparison of MVD in metastatic tumor nodules between treatment group and control group. Each group contained 16 rats, *P<0.05.

In order to explore the underlying mechanism of FTY720 on suppressing tumor metastasis, we examined the population of the circulating EPCs at different time points after liver resection and I/R injury. Before the major hepatectomy and partial I/R injury, the number of circulating EPCs did not show notable difference between the treatment group and control group (Figure 2A). The number of circulating EPCs was reduced at day 1and day 3 after the major hepatectomy and partial I/R injury in both groups. For both treatment and control groups, the level of circulating EPCs was higher in the rats with metastasis than without metastasis. The average level of circulating EPCs was significantly decreased by FTY720 treatment from day 7 to day 28 (day7: 54 *vs* 295/10^5^ PBMC cells, *P = 0.001*; day14: 46 *vs* 607/10^5^ PBMC cells, *P = 0.000*; day21: 48 *vs* 672/10^5^ PBMC cells, *P = 0.000*; day28: 124 *vs* 2345/10^5^ PBMC cells, *P = 0.000*) (Figure 3A). Furthermore, at four weeks after liver resection and I/R injury, the number of EPCs in bone-marrow in treatment group was also less than the control group (22.5 *vs* 83.6/10^5^ Cells, *P = 0.000*) (Figure 2B).

### FTY720 decreased the MVD in metastatic tumor nodules

We analyzed the expression of CD34 in intrahepatic and lung metastatic tumor nodules by IHC staining. At four weeks after tumor resection and I/R injury, immunostaining revealed a large number of CD34 positive cells in intrahepatic and lung metastatic tumor nodules in the control group. On the contrary, only a few number of CD34 positive cells were detected in the treatment group (Figure 3A). Furthermore, MVD was also decreased in both intrahepatic and lung metastatic tumor nodules after FTY720 treatment (Figure 3B).

### FTY720 attenuated hepatic architectural damage and apoptosis

**Figure 4:**
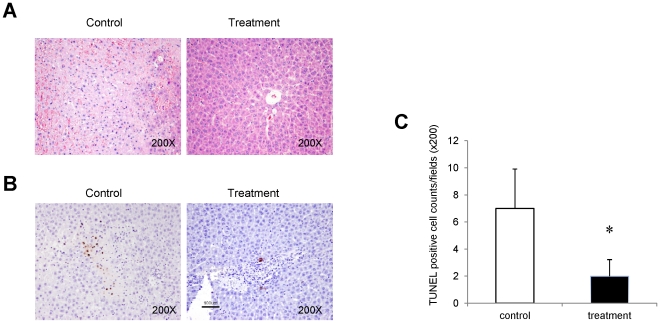
Hepatic architecture and apoptosis at 6 hours after hepatectomy and hepatic I/R injury. (A) Hepatic architecture was examined by H&E staining. (B) Hepatic apoptosis was detected by TUNEL staining. (C) Comparison of average apoptotic positive cells in 6 random fields (200×). Each group contained 6 rats, *P<0.05.

Although with slight lymphocyte infiltration, hepatic lobular architecture was well preserved after FTY720 treatment at 6 hours after reperfusion (Figure 4A). The hepatocytes and portal tracts showed normal morphological features. On the contrary, damage of vascular endothelial cells together with patchy necrosis was found around the portal tract in the control group at 6 h after reperfusion (Figure 4A). Furthermore, FTY720 also prevented hepatic apoptosis at 6 hours after reperfusion. Most of the apoptotic liver cells were found in the control groups. However, only a few apoptotic liver cells were found in the FTY720 treatment group. (Figure 4B, C).

### FTY720 down-regulated the expression of CXCL10, VEGF, CXCR3, and CXCR4

**Figure 5:**
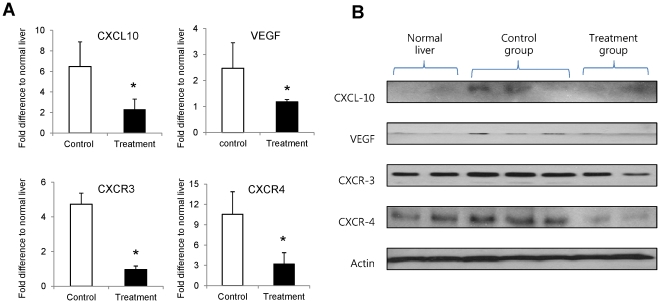
Hepatic gene and protein expressions at 6 hours after hepatectomy and hepatic I/R injury. (A) Hepatic mRNA expressions by real time RT-PCR in different groups. (B) Hepatic protein expressions by Western blot. *P<0.05.

In order to explore the underlying mechanism of FTY720 on suppression of circulating and bone marrow EPCs, the mRNA and protein expression levels of CXCL10, VEGF, CXCR3, and CXCR4 in liver tissue were compared between control group and treatment group. FTY720 significantly down-regulated mRNA and protein expression levels of CXCL10, VEGF, CXCR3, and CXCR4 in liver tissue at 6 hours after major hepatectomy and hepatic I/R injury compared to control group (Figure 5).

## Discussion

Surgical stress injury such as hepatic I/R injury during liver resection and liver transplantation rapidly enhances the number of circulating EPCs [5]–[7]. EPCs play an important role in tumor growth and metastasis [15]–[18], [20]. Therefore, it is worthwhile to study whether FTY720 suppresses liver tumor metastasis after surgical resection by attenuating hepatic I/R injury and reducing circulating EPCs.

In the present study, the effect of FTY720 on inhibition of liver tumor metastasis after liver resection and I/R injury was first shown in an orthotopic rat liver tumor model with local and distant metastatic potentials. This model effectively mimicked clinical liver tumor metastasis situation after liver surgery. In addition to reduce the incidence of tumor metastasis, FTY720 also significantly decreased the number and size of metastatic tumor nodules at four weeks after liver resection and hepatic I/R injury.

In this study, our results showed that FTY720 significantly reduced the number of circulating and bone marrow EPCs. The phenomenon was consistent with the decrease of MVD in metastatic tumor nodules. EPCs play important roles in tumor vasculogenesis and tumor growth at early phase [10], [15]–[17]. Our results also confirmed that in both treatment and control groups, the level of circulating EPCs was higher in the rats with metastasis than without metastasis. Several studies in mice and human have demonstrated that EPCs derived from bone marrow contribute to tumor angiogenesis [15], [16], [31]. The extent of the contribution depended on the tumor type, host and stage of tumorigenesis [32]. EPCs ablation can result in significant angiogenesis inhibition and impaired tumor growth and metastasis [18], [33], [34]. Furthermore, EPCs have major roles in tumor progression from micrometastases to macrometastases [18]. Therefore, the effect of FTY720 on inhibition of liver tumor metastasis was probably due to the decrease of circulating EPCs level. Targeting circulating EPCs by FTY720 treatment may effectively decrease tumor metastasis and block metastasis progression from micrometastases to macrometastases. However, the direct effects of FTY720 on the generation of EPCs in bone marrow need to be further investigated.

To investigate the underlying mechanism of FTY720 on suppression of circulating EPCs, the expression level of several inflammatory chemokines and cytokines were analyzed. Our studies indicated that FTY720 significantly attenuated hepatic I/R injury and down-regulated intracellular mRNA and protein levels of CXCL10, VEGF, CXCR4 and CXCR3. Several researches show that surgery stress injury such as I/R injury during liver resection and liver transplantation rapidly enhances the number of circulating EPCs [5]–[7]. Mobilization and migration of EPCs is also associated with elevated levels of angiogenic growth factors or chemokines [8]–[10]. Recent studies demonstrate that VEGF, CXCR3 and CXCR4 could play important roles in the migration of circulating EPCs and enhanced vasculogenesis which subsequently contribute to neovascularization [8], [35], [36]. Vascular endothelial growth inhibitor effectively inhibits mobilization and differentiation of EPCs by suppression of endothelial cell-specific gene expression in early-stage EPCs and induction of apoptosis in late-stage EPCs [37]. Moreover, CXCL10 and VEGF also play important role in tumor angiogenesis [38], [39]. Our previous study also showed that the downregulation of CXCL10 and VEGF by adiponectin treatment could effectively reduce liver tumor growth and metastasis [27]. Therefore, our result suggested suppression of CXCL10, VEGF, CXCR3 and CXCR4 expression by FTY720 may be one of the mechanisms contributing to the reduction of circulating EPCs and the decrease of neoangiogenesis. The specific mechanisms of these molecules on FTY720-mediated suppressions of circulating EPCs and neoangiogenesis need to be further clarified.

In conclusion, FTY720 suppressed liver tumor metastasis after hepatectomy and I/R injury through attenuating hepatic I/R injury and reducing the numbers of circulating EPCs, suggesting that it may be a promising candidate for potential adjuvant therapies for treating liver cancer metastasis after liver surgery for HCC patients.
